# How smartphones are changing the face of mobile and participatory healthcare: an overview, with example from eCAALYX

**DOI:** 10.1186/1475-925X-10-24

**Published:** 2011-04-05

**Authors:** Maged N Kamel Boulos, Steve Wheeler, Carlos Tavares, Ray Jones

**Affiliations:** 1 Faculty of Health, University of Plymouth, Drake Circus, Plymouth, Devon PL4 8AA, UK; 2 Faculty of Education, University of Plymouth, Drake Circus, Plymouth, Devon PL4 8AA, UK; 3 Information and Communication Systems Unit, INESC PORTO, Campus da FEUP, Rua Dr. Roberto Frias, 378, 4200-465 Porto, Portugal

## Abstract

The latest generation of smartphones are increasingly viewed as handheld computers rather than as phones, due to their powerful on-board computing capability, capacious memories, large screens and open operating systems that encourage application development. This paper provides a brief state-of-the-art overview of health and healthcare smartphone apps (applications) on the market today, including emerging trends and market uptake. Platforms available today include Android, Apple iOS, RIM BlackBerry, Symbian, and Windows (Windows Mobile 6.x and the emerging Windows Phone 7 platform). The paper covers apps targeting both laypersons/patients and healthcare professionals in various scenarios, e.g., health, fitness and lifestyle education and management apps; ambient assisted living apps; continuing professional education tools; and apps for public health surveillance. Among the surveyed apps are those assisting in chronic disease management, whether as standalone apps or part of a BAN (Body Area Network) and remote server configuration. We describe in detail the development of a smartphone app within eCAALYX (Enhanced Complete Ambient Assisted Living Experiment, 2009-2012), an EU-funded project for older people with multiple chronic conditions. The eCAALYX Android smartphone app receives input from a BAN (a patient-wearable smart garment with wireless health sensors) and the GPS (Global Positioning System) location sensor in the smartphone, and communicates over the Internet with a remote server accessible by healthcare professionals who are in charge of the remote monitoring and management of the older patient with multiple chronic conditions. Finally, we briefly discuss barriers to adoption of health and healthcare smartphone apps (e.g., cost, network bandwidth and battery power efficiency, usability, privacy issues, etc.), as well as some workarounds to mitigate those barriers.

## Introduction

**Figure 1:**
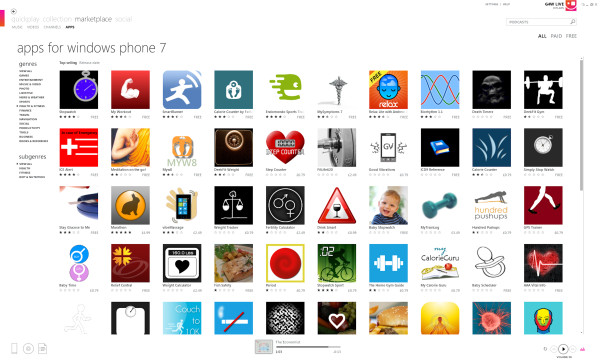
**Screenshot of Windows Phone 7 Marketplace, showing some of the health and healthcare-related apps that are currently available for downloading, including many free ones (see **[3]**)**.

More than half of Americans aged 25-29 now live in households with mobile phones but no traditional landline telephones, a December 2010 report on phone use by the US National Center for Health Statistics at the CDC (Centers for Disease Control and Prevention) has found. The same study also found that the younger children are, the likelier they are to live in homes that only have wireless phones, suggesting that younger parents are becoming increasingly reliant on mobile phones even as they adjust from being single to a more settled family lifestyle [1]. According to a recent video report by Mobile Future, a Washington, D.C., broad-based coalition of businesses and non-profit organisations, there has been a massive increase in the numbers of consumer smartphone apps (applications) downloaded over the past two years, with figures going up from 300 million apps downloaded in 2009 to five billion in 2010 [2,3] (Figure 1).

Indeed, smartphones have been one of the success stories of the last decade. In a relatively short period of time, smart mobile technology has penetrated significantly into society, capturing an entire age spectrum of subscribers in western industrialised nations, from school children to senior citizens. Such progress has built upon a long history of the use of communication devices, and a rapid adoption of mobile communication devices that began in the latter part of the last century.

According to Traxler [4] such rapid uptake in mobile phone ownership has transformed many aspects of our lives, both in the Western world and just about everywhere else around the globe. It is impacting, he suggests, not only on the manner in which we communicate, but also on our sense of culture, community, identity and relationships. Although encounters via mobile telephony are generally briefer than face to face interactions, there is evidence that for young people in particular, the number of daily contacts through text messaging can be very high [5]. Many older people also use mobile phones on a regular basis, to sustain contact with distant relatives and friends, and to converse on a daily basis, helped by call costs being generally distance independent. However, the mobile phone can undoubtedly be viewed as much more than a simple communication device [6]. It exerts a far reaching influence in society, because in effect, the mobile phone has enabled us to become 'distributed beings', due to the fact that mobile communication has unfettered us from our geographical boundedness [7]. Mobile phones appear therefore to be at the vanguard of a cultural shift where users are encouraged to constantly seek out new information and make connections with increasingly dispersed media content [8]. Whilst the demographic statistics may vary from country to country, the smartphone is a phenomenon that is here to stay, and one which will rapidly progress in its evolution in the years to come. There is therefore great scope to harness the potential of mobile telephony to improve many aspects of society, including healthcare.

### On smartphones

Although the mobile phone has been widely used for several decades, smartphones are a more recent advance. They are mobile phones that offer not only the standard facilities such as voice and text communication, but also advanced computing and communication capability, including, for example, Internet access and geo-positioning systems. In comparison to earlier mobile phones, smartphones generally also have larger, higher resolution display screens. Most of the newer generation of smartphones also incorporate other features such as on-board personal management tools, high quality cameras and recording devices.

Some smartphones, such as the Blackberry, also incorporate small internal keyboards in their designs. Recently, Apple's iPhone and Google's Android touch screen devices have increased smartphone ownership. They are popular because of their intuitive and tactile graphical user interfaces and natural gesture control. The latest generation of smartphones are increasingly viewed as handheld computers rather than as phones, due to their powerful on-board computing capability, capacious memories, large screens and open operating systems that encourage application development [9]. The potential for the creation of simple and easy to download apps for smartphones has created a vibrant new industry. There is now an app for just about every social, entertainment and educational requirement [2].

Smartphones have now achieved such a pervasive presence in society that users find it easy to self-organise themselves across large geographical areas [10]. Many have adopted a culture where they are 'always connected' to their peer groups, communities of practice and information [11]. The mobile phone provides an essential 'any time, any place' portal into the entire world wide web of knowledge. Such continuous and pervasive social connectivity has important implications for society, and holds a lot of potential in particular for use in education, healthcare and medicine.

## Mobile phone applications in healthcare

It is clear that the potential for mobile communication to transform healthcare and clinical intervention in the community is tremendous. Several previous studies have evaluated the use of mobile phones to support healthcare and public health interventions, notably in the collection and collation of data for healthcare research [12], and as used in support of medical and healthcare education and clinical practice in the community [13]. Some studies have highlighted the successful use of mobile phones to support telemedicine and remote healthcare in developing nations [14], with examples including their use in off-site medical diagnosis [15] and as information support in the treatment of HIV care in difficult to reach rural areas [16].

Studies assessing specific functionalities of smartphones have recently featured in the literature, including an examination of the use of on-board digital diaries in symptom research [17], the use of short message service (SMS) text in the management of behaviour change [18], in sexual health education [19], and to improve patients' adherence to antiretroviral treatment [20]. One study compared the use of mobile phone records against traditional paper based records in controlled drug trials [21].

GPS (Global Positioning System) and location-enabled smartphones offer many additional application opportunities that can further assist the independent living of persons with disabilities and/or multiple chronic conditions [22,23], as well as in epidemiology/public health surveillance and community data collection [24].

Kailas et al. [25] claim that there are already in excess of 7,000 documented cases of smartphone health apps. Extensive reviews of the use of mobile phone and handheld computing devices in health and clinical practice can be found by Free et al. [26] and Terry [27]. Free et al. [26] highlight several key features that give mobile phones the advantage over other information and communication technologies, including portability, continuous uninterrupted data stream, and the capability through sufficient computing power to support multimedia software applications. Significant economic benefits have also been reported where mobile communication is employed in the provision of remote healthcare advice and telemedicine [28].

### The eCAALYX example

The eCAALYX Mobile Application is being developed under the scope of the eCAALYX EU-funded project (Enhanced Complete Ambient Assisted Living Experiment, 2009-2012; [29,30]), which aims at building a remote monitoring system targeting older people with multiple chronic diseases. Patients, carers and clinicians' involvement is extensive throughout the prototype design, deployment and testing, and clinical trial phases of the project. The main functionality of the eCAALYX Mobile Platform is to act as a seamless "informed" intermediary between the wearable health sensors (in a 'smart garment') used by the older person and the health professionals' Internet site, by reporting to the latter (but also to the patients) alerts and measurements obtained from sensors and the geographic location (via smartphone GPS) of the user. Additionally, the mobile platform is also able to reason with the raw sensor data to identify higher level information, including easy-to-detect anomalies such as tachycardia and signs of respiratory infections, based on established medical knowledge. A user interface is also provided, which allows the user to evaluate the most recent medical details obtained from sensors, perform new measurements, and communicate with the caretakers.

#### Major challenges

There are many challenges to the development of the mobile platform. Most importantly, the mobile platform must be seamless and autonomous in its operation (e.g., in raising alerts), in order to provide a usable service to a target group that usually does not have any familiarity with technology and might even be unconscious during times of medical emergency and not able to manually operate any device or software. System and service reliability is also an important issue to take into account, firstly due to the possible negative sensation that the application may give to the user in the case of malfunctioning, and, secondly, due to the physical distance between the technical maintenance teams and the users. From an implementation point of view, the issues regarding the implementation of intelligent mechanisms in a mobile resource-limited device should also be considered. Usability issues are further discussed in the following section.

##### Usability

Usability is a critical issue for the target group of eCAALYX as, usually, users in this target group do not have any familiarity with technology and this is also often compounded by a range of physical (e.g., poor eyesight) and/or cognitive disabilities (e.g., dementia) that such users might be suffering from that can further limit their use of the technology [23]. Due to that fact, the eCAALYX Mobile Platform was designed to be completely transparent to the user, and the necessary interface functionality to be as accessible as possible. The mobile platform and some of eCAALYX software functionalities are depicted in Figure 2 and a video walkthrough available from [31]. The usability design for this mobile platform caters for older users' needs in two main areas, namely physical handling and maintenance of the smartphone, and the usage of the phone software itself. Regarding physical handling and maintenance, the following practical solutions were adopted:

**Figure 2:**
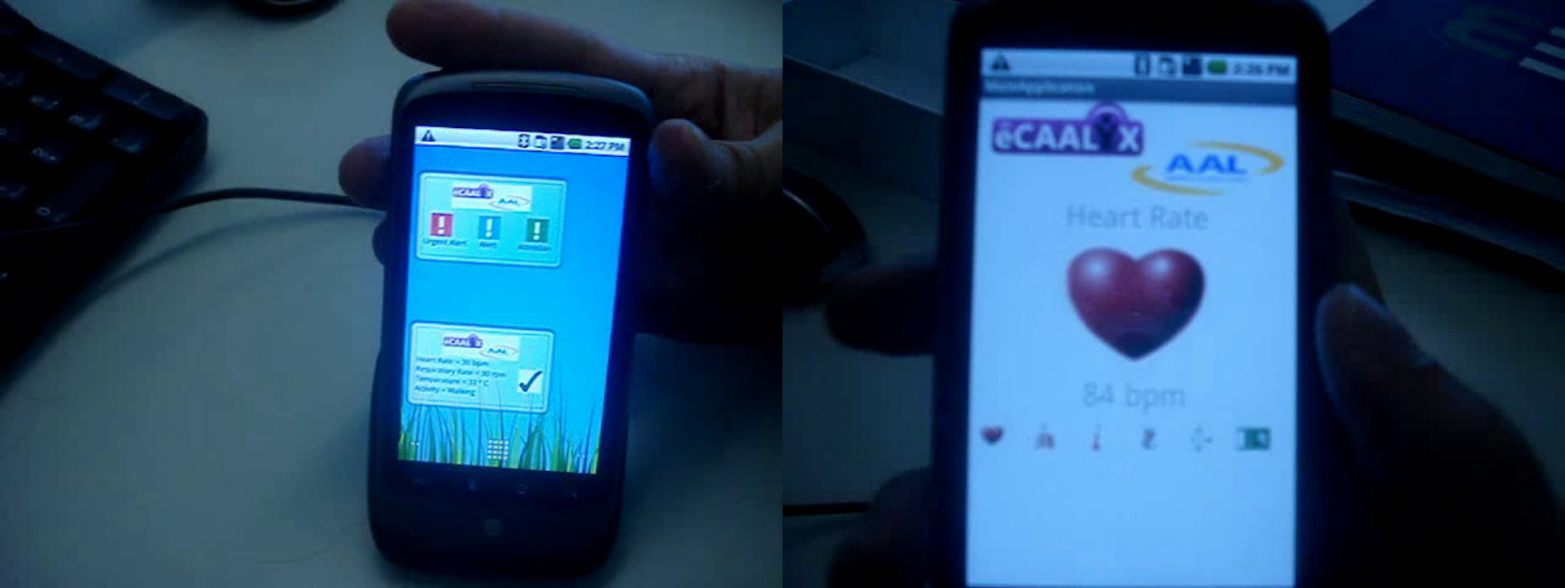
**Left: Main screen of the eCAALYX Mobile Application; Right: Visualisation of the heart rate measurement**. A video walkthrough of the eCAALYX Mobile App is available from [31].

• Use of dock-stations to simplify the battery charging of the mobile device;

• Use of a mobile phone without buttons and with large touch-screens, which allows the building of virtual buttons as large as needed, instead of the small buttons available on commercial mobile phones with conventional keypads and keyboards; and

• All maintenance actions are performed either remotely and transparently to the user, or locally, by technicians.

Concerning smartphone software usage, the following practical solutions were adopted:

• The phone runs autonomously without the need of any mandatory interaction from the user from the time it is powered on. This includes the supressing of all enquiries of the operating system, such as pin negotiation and the automation of all necessary processes;

• Rebooting has to be avoided, because it can be a difficult task to perform by the target users. The phone must therefore support prolonged periods of operation without the need to reboot the system;

• All possible navigation has been reduced to two easily accessible screens, to avoid confusion of the user while navigating the software; and

• All error pop-ups were supressed, to avoid showing any system errors to the user.

#### Prototype

**Figure 3:**
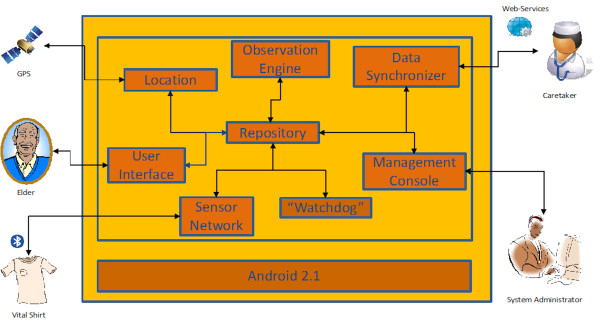
**Architecture of the eCAALYX Mobile Platform**.

The technological platform in the current prototype is the Google Nexus, running the Android 2.1 platform [32], with 1 GHz processor and 512 MB RAM memory; however, it can be easily ported to a newer Android version, or even to another Android phone. The software itself is written in the JAVA language for the Android's Dalvik Virtual Machine. From a software point of view, the internal structure follows a black-board architecture, in which several concurrent processes share information using the SQLite database provided in the Android Platform. Access to necessary resources, such as GPS, Bluetooth, and the Internet, is also provided through the Android Platform. The interface with the Caretaker/Clinicians' site is accomplished using the W3C Web Services technology, while the interface with the health sensors (in a 'smart garment' worn by the patient) is realised using Bluetooth wireless technology. This structure is shown in Figure 3.

There are not yet any clinical trials or evaluation results of the eCAALYX Mobile App to report (at the time of writing in January 2011); such details are expected to be available after project completion in July 2012. However, we think that the eCAALYX Mobile App we have briefly described here provides a unique opportunity to have a somewhat detailed 'under-the-hood' look at one current example from a rapidly growing class of smartphone apps for chronic disease management and telehealthcare.

## Discussion

eCAALYX builds on the experience gained in a previous and closely related EU-funded project, CAALYX (Complete Ambient Assisted Living Experiment, 2007-2008; [23,30,33]). The original CAALYX prototype similarly used a smartphone and one of the prototype's commercial exploitation barriers identified at that time was the relatively high cost of acquiring an Internet- and GPS-enabled smartphone with sufficient computing power (and battery life) to run the CAALYX mobile app (plus the subscription costs to a suitable mobile phone data plan), particularly given the generally low income of target users (older pensioners [34]). However, recent surveys of mobile phone uptake and penetration in the UK and other developing countries show that this affordability barrier might be gradually improving on the medium to long run, especially for the "younger" older generations (55-70) and as smartphone prices and data plan costs continue to drop.

### A closer look at ownership of smartphones

Smartphone ownership in the developed world is rapidly increasing. ChangeWave Research [35] report a (presumably US) survey in December 2009 with 42% saying they own a smartphone (rising from 15% in October 2006) (but details of the survey methods are not freely available). Estimates from Ofcom research [36] suggest the figure is less, namely that the USA has 36% and the UK has 37% with Internet access via a mobile phone. Ofcom also cites ComScore's *MobiLens *survey [37] estimating only 18% of people with smartphone subscriptions in the UK compared to 26% in Italy and 21% in Spain. Ofcom reports that there is a good deal of consumer confusion over the capabilities of their phone but that 43% claim their phone can be used to access apps, download email, and surf the Web. If 81% have a mobile phone (see below) this would imply a third of the UK population with smartphones. We might estimate therefore that between one fifth and one third of the UK population now has a smartphone.

### What are the predictions for smartphone ownership to reach over 90% of the population?

We can perhaps extrapolate from ownership of any mobile phone. UK's National Statistics [38] report the growth in mobile phone ownership since 2001/02, increasing from 65% to 81% in 2009. Ownership varies by income group. Only 67% of households in the lowest income decile group reported mobile phone ownership in 2009, compared with 92% in the highest income decile group. (Recent estimates of mobile phone use in the USA are 85% [39], so there is no significant difference between UK and USA (given the differences in methods used and error in these estimates).)

**Figure 4:**
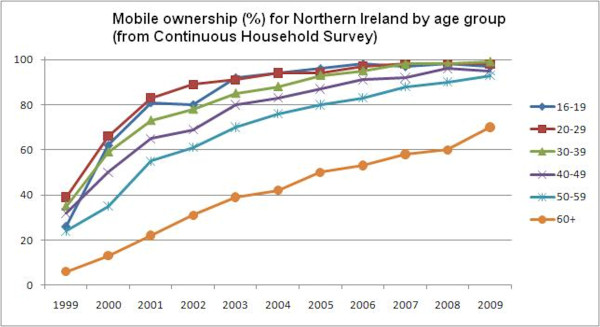
**Mobile ownership (%) for Northern Ireland by age group**. Figure constructed from data from the Northern Ireland Continuous Household Survey [40].

Age is probably the major 'digital divide'. The Continuous Household Survey in Northern Ireland [40] provides a good indication of how mobile phone ownership is increasing. It puts current ownership at 89% in Northern Ireland. It is not clear whether this higher figure compared to the British 81% from National Statistics is 'real' or due to methodological differences. Nevertheless, the pattern of older consumers lagging behind the rest of the population (Figure 4) is likely also to be the case in England and the rest of the UK. However, if the current trajectory continues, over 95% of the over 60 s will have a mobile phone in five years.

Taking these two trends together we might estimate that 80-90% of the UK population will have a smartphone within 10 years. This might not be quite as rapid as some might suggest, but, in the context of other social changes brought about by the development of technology, is a massive change.

### What impact will this have on healthcare?

Jane Sarasohn-Kahn reports "How smartphones are changing healthcare for consumers and providers" [41]. Probably the quickest change is in the use of smartphones by professionals. The slightly slower, but possibly bigger, impact may be in the use of smartphones by lay consumers. Producers of apps certainly think there is a market, as by February 2010, some 4000 apps were available within the Apple App Store aimed at patient end-users, and Gartner named mobile health as one of its top ten applications for 2012 [42].

Considering that many apps now, especially those intended for chronic disease management, rely on the presence of an active Internet connection on the smartphone in order to function as intended, a question immediately arises about current Internet uptake levels among people with chronic diseases.

### Do people with chronic disease go online and can Internet-enabled smartphones make a difference?

Fox and Purcell [43] report that in the USA, adults living with chronic disease are disproportionately offline. 81% of adults reporting no chronic diseases go online compared to 62% of adults living with one or more chronic disease. And there is a marked 'dose response', so 68% of adults reporting one chronic disease go online compared to only 52% of adults living with two or more chronic diseases. The difference is even more marked when comparing use of text messages with only 23% of those with two or more chronic conditions compared to 60% of those with no conditions using a mobile to send text messages [43].

There are now many health promoting Internet interventions [44] but they will, by definition, only be used by those who have already reached a decision to try to change their behaviour. Although many report successful behaviour change in those who continue to use such interventions, attrition is usually very high, and we can assume that those who drop out of using the online intervention have also dropped out of changing their behaviour. It may be that smartphone technology, by its mobility and location awareness, may be able to achieve lower attrition rates, but given the demographics of current users it is likely to be for behaviour change amongst a relatively young and healthy population. Sarasohn-Kahn [41] had argued that medication adherence is a problem amongst patients with chronic conditions and suggested that technology can play an important role. However, there is no strong evidence yet on the effectiveness of dispensing devices, and like behaviour change, this is likely only to benefit those willing to use such reminders and who are already smartphone users, unless apps running in specialised devices can be developed.

There are now hundreds of smartphone apps focusing on wellness, fitness, and nutrition. Mobile and home monitoring can be carried out with body-worn and ambient sensors communicating with smartphones (as found, for example, in eCAALYX), including accelerometers measuring motion and gait, infrared detectors measuring body temperature and heart rate, and glucometers measuring blood glucose (some sensors may also be built-into future smartphones). However, Sarasohn-Kahn [41] describes how MedApps started as a mobile phone app, but given the low user base amongst chronically ill and older people they re-engineered it as a wireless handheld device, the HealthPal. Users use their 'usual' devices such glucometers, spirometers, pulse oximeters, and scales, and the Bluetooth enabled Healthpal communicates results. Another rather obvious consideration in smartphone use for people who are (chronically) ill, is that most such people are at home, and unlikely to be 'out and about' using their mobile phone. In that circumstance they are more likely, with current device usage, to use a Wi-Fi enabled laptop (or iPad/Android Touch Tablet) from their bed (although the use of 3G mobile Internet connections indoors is also on the rise on different devices ranging from smartphones to notebooks). However, smartphones, especially newer ones with larger (touch) screens, are starting to replace conventional desktop and Wi-Fi access (and some smartphones are both Wi-Fi and 3G mobile Internet enabled). In the UK, 29% of Internet users use their mobile to access the Internet at home, second only to those in Japan at 43% [36], and mobiles are rapidly replacing landlines in many homes. In the UK, 13% of all homes used mobiles as their sole form of telephony [36], but more than half of Americans aged 25-29 now live in households with mobile phones but no traditional landline telephones [1].

At the moment, Sarasohn-Kahn [41] cites Eising of Mayo clinic mentioning their research that people use mobiles for 'action-oriented' information, and how they are not going to do in-depth research by mobile. So smartphones will be used by people who are not acutely ill but who maybe want to find some location based information - such as the location and hours of a pharmacist - while on the move. Whereas more detailed information, or communication with others may take place in the home. But as more people get smartphones and use them as their sole means of communication, this may change.

What other barriers to smartphone use in health does Sarasohn-Kahn [41] see? She is concerned that too much app development is carried out by technologists without the involvement of patients. She notes the problems of apps recommending particular products and also notes that in the USA if an app includes a sensor then the FDA (US Food and Drug Administration) may monitor it as a medical device. She thinks that the challenges to continued rapid smartphone growth include finding the right business model and privacy issues. (For a brief discussion of the latter privacy issues and some workarounds adopted in CAALYX/eCAALYX, interested readers may refer to [23] and [45]).

### Some limitations of mobile phone applications

Notwithstanding all of the benefits we should be aware that the use of the mobile phone in healthcare and clinical practice is not without its problems and limitations. In comparison to laptop computers, the small internal storage capacity, processing power and screen size of the mobile phone often requires apps that are running to be in reduced format [25]. However, the use of cloud computing resources which are external to the mobile device may obviate restrictive processing speeds and memory requirements in the future [25]. Never the less, mobile phones are smaller, more portable and less obtrusive than laptops, so it could be argued that this is a reasonable trade off. Although much mobile phone communication is now conducted using text, voice communication still necessitates the securing of space within which vocal communication can be made in private [46]. The consideration of such a constraint may be vital to maintain the confidentiality of patient information if used in public spaces. Other factors such as loss or theft of devices may impact upon the security of confidential digital health records or data held on mobile phones. The security of patient data held on mobile phones has been a concern for some time [47], while some studies warn of the security risks of using mobile instant messaging in healthcare [48].

### Patient attitudes and perceptions

When used as a method for monitoring the health status of remote patients, mobile phones should be applied only after due consideration to patient perceptions and feelings. One study in Italy revealed that patients with implantable cardioverter-defibrillators for cardiac resynchronisation therapy welcomed the use of mobile technologies for remote monitoring, but did not want them to replace their personal contact with health workers [49]. Other studies using mobile technology for remote monitoring of health conditions found similar results [50,51]. The Canadian study by Seto et al. [51] also advised that mobile phone-based remote monitoring will not be suitable for all patients. Those for example who suffer from poor manual dexterity, failing vision or a predisposition to high levels of anxiety may not be able to operate the remote mobile monitoring tools. These results are supported by similar findings from a study of older patients with disabilities in the USA [52]. It should also be acknowledged that prosaic issues such as remembering to recharge a device and the simple maintenance of equipment within a patient's home may be problematic.

Kurniawan [53], Lorenz and Oppermann [54], and Gao and Koronios [55] provide detailed discussions of mobile app needs of older people, covering aspects such as ergonomics and user interface issues (e.g., memory aids, visual aids, haptic aids, features to minimise user error, and safety features), as well as the most commonly desired smartphone app functionalities in this age group. However, senior citizens of tomorrow will include the young and middle aged of today, who are more familiar with, and reliant on computers, smartphones and the Internet than previous generations, and are increasingly well-versed in using these technologies on a daily basis for study, work and leisure. This might partially contribute (in the long run) to solving some of the smartphone app usability and learnability issues, which current generations of older people are facing.

### Which platform to support? A developer's dilemma

According to a recent MobiHealthNews report published in November 2010 [56], from February to September of the same year, Google's Android smartphone saw a 156.6% increase in the number of available health-related apps, compared with a 66.6% increase in Apple's health-related apps. The number of health-related apps in BlackBerry's App World increased by 141.4% over the same period. However, Apple is still leading in terms of the absolute total number of health-related apps available on any platform. As of September 2010, Apple's App Store offered the highest number of health-related apps at 7,136, followed by Google Android at 1,296 and BlackBerry at 338. These figures represent a healthy 78% increase in the number of health-related apps on these three platforms combined since February 2010. The MobiHealthNews report did not cover the latest Microsoft Windows Phone 7 platform as this was only officially launched during October/November 2010; older versions of the Windows platform (Windows Mobile 6.x) had less than a 3% market share of worldwide 2010 Q3 smartphone sales (vs. 25.5% for Android, 16.7% for Apple iOS and 14.8% for Research In Motion--RIM BlackBerry) [42], and were probably not considered in the MobiHealthNews report for this reason. However, the emerging Windows Phone 7 platform is rapidly gathering momentum and support by major smartphone providers [57], with a growing number of health-related apps already available from Windows Phone 7 Marketplace for the new handsets (Figure 1 - [3]). It remains to be seen how the emerging Windows Phone 7 platform will fare against the well-established competitor smartphone players and their planned updates in 2011/2012.

### Other constraints

Desk-based health researchers who rely on the telephone to gather their data are faced with a growing problem. Increasingly, respondents are replacing their landlines with mobile telephones, and in so doing, they create a problem for the researcher. Legislation in some countries requires researchers to manually dial mobile phone numbers, thereby incurring significantly more cost in both time spent on calling and in call costs. Further, calling a mobile phone on some tariffs may use up respondents' air time, and there may therefore be an ethical onus on the researcher to reimburse the costs incurred [58].

## Conclusions

It is clear from their rapid proliferation and deep penetration into society, that there are significant opportunities to exploit the potential of smartphones in healthcare [27]. Mobile health (m-health) applications are on the rise, with many clinicians and allied health workers already adopting smartphones successfully in a diverse range of practices. Patients too are accessing health information, actively participating in their own care (participatory healthcare), and maintaining contact with their healthcare providers through smartphones [25,27]. Chronic conditions such as diabetes mellitus and cardiovascular disease have in particular always been perceived as a special 'niche market' for smartphone apps [59-63].

Some commentators [27,41] suggest that the natural progression for healthcare is to go mobile, because it is information intensive and smartphones can offer a convenient solution. Smartphones are useful to keep clinicians up to date with the latest medical techniques, and it is easy and cost effective to communicate updates, advice and guidelines to a distributed community of practice in this way. As has also been demonstrated, mobile phones are useful for monitoring and diagnosing health conditions when clinicians are a distance from their patients. Further, with the Internet playing an increasing role in medical education [64], it is likely that for itinerant health workers the most important access portal to this information will be handheld devices such as smartphones. Indeed, Georgetown Medical School in the USA, for example, is now requiring every medical student to have an iPhone [65], and surgeons are finding the device (and its apps) very useful in improving their diagnostic skills and education [66]. Smartphones are therefore useful to the medical and health related professions because they are agile, handheld, easy to use and can be used on the move [41].

Later adopters of new technologies may not use them in the same way as early adopters. Developers of new smartphone health apps need to look 'at the margin', i.e., how the latest group of adopters are using smartphones and how the next group of new users may use it. Although there are hundreds of smartphone apps at the moment, the successful ones are, currently, likely to be for younger and healthier populations. The adoption of smartphones by older people and people with chronic disease will come with time, but also as the relative cost comes down, as apps become easier to use, as there is a greater awareness of what smartphones can do, with the establishment of more 'community knowledge' to deal with the complexity of the new technology [67], and perhaps with apps moved to dedicated devices tailored for the specific needs of particular user groups and applications. These changes will almost certainly happen, but probably not as quickly as producers may predict. Producers may need patience and to put more effort into making the technology easier and cheaper to use for all.

## Competing interests

The authors declare that they have no competing interests.

## Authors' contributions

MNKB conceived and drafted the manuscript with contributions from SW, CT and RJ. MNKB and CT (and their institutions) are both involved in the EU-funded eCAALYX project, with MNKB leading the University of Plymouth part of it. All authors read and approved the final version of the paper. (N.B.: All Web links in 'References' below have been last checked working on 16 March 2011.)
